# Supplementary material for: Cost-Effectiveness of a Problem-Solving Intervention Aimed to Prevent Sickness Absence among Employees with Common Mental Disorders or Occupational Stress
Source: Int J Environ Res Public Health. 2020 Jul 20;17(14):5234. doi: 10.3390/ijerph17145234 (PMC7400406; doi:10.3390/ijerph17145234)
Supplement: Supplementary file 1 [file ijerph-17-05234-s001.zip › Supplementary Material 1.pdf]

Table S1. SMS messages were sent to the participants every fourth week over the follow-up period with start one month after the baseline questionnaire was completed.

Question	Response format
**SMS**	
Are you currently on sick leave?	no sick leave, 25%, 50%, 75% or 100% sick leave
Over the last four weeks, how much did your health problems affect your performance while you were working?	0 to 10; higher numbers indicate higher performance limitations
**Questionnaire**	
What are your ordinary working hours? (choose the most correct alternative)	Full-time (40h/week)<br>Part-time (75%)<br>Part-time (50%)<br>Part-time (25%)
Choose the interventions for stress, concern, depression, or other mental ill health you received during the last six months. the intervention might have been delivered by the occupational health service or any other actor/health care services. you can choose several options.	Medical doctor; psychologist/psychotherapist; welfare officer; physiotherapist; meditation/mindfulness; yoga; tai chi; Qi Gong; FaR*; acupuncture/acupressure; other, please specify

*FaR = Physical Activity on Prescription
