# Supplementary material for: Cost-Effectiveness of a Problem-Solving Intervention Aimed to Prevent Sickness Absence among Employees with Common Mental Disorders or Occupational Stress
Source: Int J Environ Res Public Health. 2020 Jul 20;17(14):5234. doi: 10.3390/ijerph17145234 (PMC7400406; doi:10.3390/ijerph17145234)
Supplement: Supplementary file 1 [file ijerph-17-05234-s001.zip › Supplementary Material 2.pdf]

Table S2. Other interventions that employees received beside PSI or CAU at baseline, 6-months and 12 months

	**Baseline**				**6-months**				**12-months**			
	**PSI**		**CAU**		**PSI**		**CAU**		**PSI**		**CAU**	
	n	%	*n*	%	*n*	%	*n*	%	*n*	%	*n*	%
Medical doctor	24	59	30	51	22	65	40	77	9	27	23	46
Psychologist	15	37	23	39	22	65	37	71	10	30	24	48
Welfare officer	8	20	5	9	11	32	10	19	5	15	3	6
Physiotherapist	4	10	7	12	4	12	10	19	2	6	4	8
Alternative	14	34	10	17	13	38	28	54	8	24	28	56
Other	7	17	10	17	4	12	9	17	8	24	9	18
No co-interventions	12	31	20	35	3	9	3	6	15	46	17	34

PSI = problem-solving intervention; CAU = care as usual
Alternative: e.g. meditation, yoga and acupuncture.
Other: e.g. massage, course in stress-management, medication, relaxation exercises.
